# Exploring the multidimensional heterogeneities of glioblastoma multiforme based on sample-specific edge perturbation in gene interaction network

**DOI:** 10.3389/fimmu.2022.944030

**Published:** 2022-08-29

**Authors:** Jianglin Zheng, Yue Qiu, Zhipeng Wu, Xuan Wang, Xiaobing Jiang

**Affiliations:** ^1^ Department of Neurosurgery, Union Hospital, Tongji Medical College, Huazhong University of Science and Technology, Wuhan, China; ^2^ Department of Otolaryngology, Union Hospital, Tongji Medical College, Huazhong University of Science and Technology, Wuhan, China

**Keywords:** glioblastoma multiforme, gene interaction network, edge perturbation, prognosis, immune landscapes

## Abstract

Glioblastoma multiforme (GBM) is the most malignant brain cancer with great heterogeneities in many aspects, such as prognosis, clinicopathological features, immune landscapes, and immunotherapeutic responses. Considering that gene interaction network is relatively stable in a healthy state but widely perturbed in cancers, we sought to explore the multidimensional heterogeneities of GBM through evaluating the degree of network perturbations. The gene interaction network perturbations of GBM samples (TCGA cohort) and normal samples (GTEx database) were characterized by edge perturbations, which were quantized through evaluating the change in relative gene expression value. An unsupervised consensus clustering analysis was performed to identify edge perturbation-based clusters of GBM samples. Results revealed that the edge perturbation of GBM samples was stronger than that of normal samples. Four edge perturbation-based clusters of GBM samples were identified and showed prominent heterogeneities in prognosis, clinicopathological features, somatic genomic alterations, immune landscapes, and immunotherapeutic responses. In addition, a sample-specific perturbation of gene interaction score (SPGIScore) was constructed based on the differently expressed genes (DEGs) among four clusters, and exhibited a robust ability to predict prognosis. In conclusion, the bioinformatics approach based on sample-specific edge perturbation in gene interaction network provided a new perspective to understanding the multidimensional heterogeneities of GBM.

## Introduction

Glioblastoma multiforme (GBM) is the most malignant brain cancer with an extremely dismal prognosis despite aggressive therapeutic strategies consisting of surgical excision and chemoradiotherapy (1). GBM is characterized by high heterogeneity, including inter- and intra-tumoural heterogeneity, which pose a daunting challenge to judgment in prognosis and effective treatment (2). Searching for valuable molecular markers has long been recognized as a well-established means for the resolution of heterogeneity. With the broad application of high-throughput sequencing technology and bioinformatics analysis, increasing classifications of GBM that rely on gene expression files have been proposed (3–5). It is important to note, however, such classification approaches overlooked the dynamic nature of gene expression.

From a dynamic point of view, the gene expression in a biological system varies over time and under different conditions (6). In other words, the molecular composition of cancer cells may be different under different time points or conditions. Therefore, the gene expression profiles obtained at specific time points or conditions are not completely reliable to characterize the biological status of individual patients. In comparison, biological networks are relatively stable against time and conditions (7, 8). It has been recognized that specific biological networks, but not individual molecules, are ultimately responsible for cancer biology (9). Thus, applying network-based methods to the analysis may contribute to a better understanding of cancer heterogeneity.

In this study, we used sample-specific edge perturbation in the gene interaction network to explore the heterogeneity of GBM. This method is entirely distinct from the previous gene expression-based approaches. Two key information, gene sets (nodes in networks) and interactions (edges in networks) were utilized in this method. It has been revealed that the gene interaction network is widely perturbed in tumor tissues compared with that in normal human tissues (8). This method can quantify these perturbations in gene interactions (edge perturbations) by evaluating the change in the relative gene expression value. The edge perturbations can efficiently characterize the perturbations of the biological network for each sample. Based on the edge-perturbation matrix, we performed an unsupervised consensus clustering analysis to establish the edge perturbation-based clusters of GBM samples. Moreover, we constructed a sample-specific perturbation of gene interaction score (SPGIScore) which exhibited a robust ability to predict prognosis. Our findings are helpful to understand the heterogeneity of GBM from the perspective of biological networks.

## Methods

### Data sources and preprocessing

This sample-specific edge perturbation project was carried out based on the data mining of public databases. A total of 155 GBM samples from the Cancer Genome Atlas (TCGA) were defined as the experimental cohort, the transcriptome data and clinical information of which were downloaded from the UCSC Xena website (https://xenabrowser.net/datapages/). For the control cohort, the transcriptome data of 1152 normal brain samples were obtained from the Genotype-Tissue Expression database (GTEx, https://gtexportal.org/home/). In addition, three independent validation cohorts were composed by the GBM samples extracted from the Chinese Glioma Genome Atlas (CGGA, http://www.cgga.org.cn/; mRNA-array_301 dataset and mRNAseq_325 dataset) and Rembrandt microarray dataset (http://gliovis.bioinfo.cnio.es/). In order to maintain data consistency, the transcriptome data from different sources were converted to TPM form. Genes with zero expression in more than 70% samples were filtered out from each cohort, and batch correction was performed *via* “ComBat” function in R packet “sva”. The sample number of each cohort was presented in **Table S1**.

### Construction of background interaction network

Reactome (http://www.reactome.org) was an expert-authored and peer-reviewed biomolecular pathway database, aiming to provide bioinformatic tools for visualization and interpretation of a network of biological interactions (10). The core unit of the Reactome data mode is the reaction of different entities, including nucleic acids, proteins, complexes, anticancer therapeutics and small molecules (11). Thus, Reactome is a good choice. We used the ReactomeFIPlugIn of Cytoscape to download all gene interactions of Reactome pathways, and merged them into a large background interaction network. Specifically, this background interaction network is a gene interaction network based on Reactome pathways, including protein–protein interactions, gene coexpression, protein domain interactions, Gene Ontology (GO) annotations and text-mined protein interactions.

### Construction of the edge-perturbation matrix

As shown in the previous study (11), the construction of edge-perturbation matrix (EPM) mainly includes three steps:

Firstly, according to the expression levels of genes in the background interaction network, we obtained the rank of genes in GBM samples and normal samples, respectively. The rule is that the lower expression level corresponds to the smaller rank, and the higher expression level corresponds to the greater rank. The expression matrix was then transformed to a rank matrix.

Secondly, we refer to the interaction relationship of gene pairs in the background interaction network. If two genes interact with each other, there will be an edge connecting these two genes in the network. We then calculated the rank difference of this edge in two genes, and further obtained the delta rank matrix whose rows and columns represented edges in the background interaction network and samples, respectively. The delta rank was calculated as follows:


$$\delta_{e,\;s}=\;r_{i,\;s}-r_{j,\;s}$$

where *r_i, s_* denotes the rank of gene *i* in sample *s*. *δ_e, s_* denotes the delta rank of edge *e* in sample *s*. Gene *i* and gene *j* are connected by edge *e*.

Thirdly, it was agreed that the gene interaction of normal samples is highly conserved, while the interaction perturbations were more frequent in cancer samples (12). Next, we constructed the rank matrix based on the mean expression values of genes in normal samples and calculated the delta rank in the same manner. The delta rank of normal samples was set as the benchmark delta rank vector ( ${\bar{\delta }}_{e}$ ), which represents the average relative ranks of gene pairs in all normal samples. Hence, the gene interaction perturbations of samples can be measured by compared their delta rank with the benchmark delta rank vector. Finally, the EPM was constructed with the element Δ*_e, s_* of each sample, which was calculated as follows:


$$\Delta_{e,\;s}=\delta_{e,\;s}-{\bar{\delta }}_{e}$$

where Δ*_e, s_* represents the perturbation of edge *e* in sample *s*. Each column of the EPM represents the edge perturbations of an individual sample. Then, we could take advantage of the EPM to perform clustering analysis of GBM samples.

### Clustering analysis of GBM samples based on feature edges

To get meaningful clusters of GBM samples, we performed the unsupervised consensus clustering analysis based on the selected feature edges, which had the capacity to easily distinguish GBM samples from normal samples and didn’t lose the heterogeneity within GBM samples. By using the Kruskal–Wallis test, we first selected the top 60000 significantly different edges between GBM samples and normal samples. Next, we calculated the standard deviation (SDs) of the edge perturbations of all GBM samples and identified the top 60000 edges with high SDs. Taking the intersection of two parts, we finally obtained the GBM sample matrix with 21754 feature edges containing 4402 genes. By using R packet “ConsensusClusterPlus”, the clustering analysis was carried out based on these feature edges. The clustering distance was Euclidean and the clustering algorithm was PAM. One thousand repetitions were conducted to guarantee the stability of the cluster outcomes.

To verify the clustering performance of edge perturbation, we repeated the same procedure in a validation cohort (CGGA-mRNA-array_301). The in-group proportion (IGP) of each cluster was calculated to evaluate the consistency between clusters derived from two independent cohorts (13). A larger IGP value indicates a higher consistency between clusters. Given that the transcriptome data of TCGA cohort and CGGA-mRNA_array_301 cohort were generated in different ways, which were RNA-seq and microarray, respectively, we normalized the edge-perturbation value to Z-score before IGP analysis. The IGP analysis was performed by using R packages “clusterRepro”.

### Characteristic analysis of edge perturbation-based clusters

In order to explore the heterogeneity characteristic among edge perturbation-based clusters, we obtained multidimensional characteristic parameters of GBM samples of TCGA cohort from relevant published studies. The tumor purity and ploidy information of GBM samples were derived from a Pan-Cancer analysis project of TCGA database (14). The stemness features of GBM samples were evaluated *via* the mRNA expression-based stemness index (mRNA-si) and the epigenetically regulated-mRNAsi (EREG-mRNAsi), which were developed *via* the one-class logistic regression (OCLR) machine learning algorithm (15). Unsupervised transcriptome analysis additionally identified four transcriptomic subtypes, referred to as classical, mesenchymal, neural, and proneural, which were closely correlated with genomic abnormalities (16). Three important indicators, namely large-scale transition (LST) score, telomeric allelic imbalance (TAI) score and loss of heterozygosity (LOT) score, were used to assess the chromosomal instability levels (17). Homologous recombination deficiency (HRD) score and neoantigen load were extracted from a previous study about the immune landscape of cancers in TCGA database (18).

Additionally, some characteristic data was produced by running corresponding algorithms or with the aid of online tools. The infiltration levels of 22 types of immune cells were measured *via* the CIBERSORT algorithm. The Tumor Immune Dysfunction and Exclusion (TIDE) algorithm was performed online (http://tide.dfci.harvard.edu/) to assess the potential response to immune checkpoint inhibitor (ICI) therapy. Patients with lower TIDE scores were more likely to show better responses to ICI therapy. By using R package “Maftools”, we analyzed and visualized the somatic mutation profiles among edge perturbation-based clusters. The tumor mutation burden (TMB) was calculated as mutations per megabase (mut/Mb). The PreMSIm algorithm (19) was used to obtain the microsatellite instability (MSI) status of GBM samples, which were categorized as MSI-High (MSI-H), MSI-Low (MSI-L) and microsatellite stability (MSS). By using the GISTIC2.0 model on GenePattern (https://cloud.genepattern.org/gp/pages/index.jsf), we compared the copy number variation (CNV) frequency and presented the distribution of CNV regions among edge perturbation-based clusters.

### Cluster-specific pathway enrichment analysis

By using the Z-score method, we normalized the EPM of GBM samples to a matrix with a mean of zero for each row to zero and a variance of one. Then, we performed the following steps to screen genes used for pathway enrichment analysis. First, the normalized feature edges were clustered hierarchically by the complete linkage method. The number of groups was set to 100, and the groups with less than 30 feature edges were filtered out. Second, we calculated the percentage of the feature edges whose absolute value of perturbation mean was greater than 0.5 in all feature edges in each retained group. Finally, the group with a percentage greater than 0.7 was a characteristic group of edge perturbation-based cluster. All the genes contained in the corresponding feature edges of the characteristic groups were used for pathway enrichment analysis, which was performed on Metascape (http://metascape.org). We retained the KEGG and Reactome pathways with *P* value less than 0.01.

### Constructing the sample-specific perturbation of gene interaction score

By using the R package “limma”, the pairwise comparison was performed among edge perturbation-based clusters to identify the hub differently expressed genes (DEGs). The intersection of DEGs was utilized for subsequent univariate Cox regression analysis. Genes with *P* value less than 0.05 were regarded as the prognostic genes and were incorporated into the least absolute shrinkage and selection operator (LASSO) Cox regression by using the R package “glmnet”. The SPGIScore was consequently constructed by selecting the optimal penalty parameter λ correlated with the minimum 10-fold cross-validation. The calculation formula of SPGIScore is shown below:


$$SPGIScore=\sum_{i=1}^{n}Coef_{i}*x_{i}$$

where *x_i_* and *Coef_i_* represent the expression level of each selected gene and corresponding coefficient, respectively. The median SPGIScore was used as the cut-off value for the high/low-SPGIScore grouping. The prognostic value of SPGIScore was tested in multiple cohorts of GBM samples.

### Cell lines and tissue samples

The normal human astrocyte HA1800 cell line (HA) and human GBM U87, A172, LN229, U251 and U373 cell lines were purchased from Cell Bank of the Chinese Academy of Sciences. The cells were cultured in DMEM medium (Corning, USA) containing 1% penicillin/streptomycin (Gibco, USA), and 10% fetal bovine serum (Gibco, USA), at a condition of 37°C with 5% CO_2_. Fifteen clinical samples from GBM patients were collected from July 2020 to October 2021 at the Neurosurgery Department of Wuhan Union Hospital. In addition, ten cases of normal brain tissues (resected from surgery in patients with acute traumatic brain injury) were collected as the control group. The GBM tissues and non-tumor brain tissues (NBT) obtained from the patients were immediately frozen into the liquid nitrogen, followed by storing at -80°C before further analysis. This study was approved by the Ethics Committee of the hospital, and written informed consent was obtained from each patient.

### Quantitative real-time polymerase chain reaction (qRT-PCR) and immunohistochemistry (IHC)

The total RNAs were extracted by TRIZOL reagent (Ambion, USA) from the tissues and cells. cDNA was synthesized by reverse transcription using HiScript^®^ III RT SuperMix for qPCR (+gDNA wiper) (Vazyme, China) as the manufacturer’s instruction. The qRT-PCR was carried out by applying AceQ^®^ qPCR SYBR Green Master Mix (Vazyme, China). All expression data was normalized to β-actin as an internal control using the 2^–ΔΔCt^ method. The experiments were independently repeated at least three times. All primers used were chemically synthesized by GeneCreate Biological Engineering Co. Ltd. (Wuhan, China). Specimen tissues were formalin fixed, paraffin embedded and sectioned into 4-µm serial sections. The specimen slices were dewaxed, then hydrated and boiled in citrate buffer (pH=6) for 8 minutes to recover the antigen. Subsequently, the sections were treated to quench endogenous peroxidase activity. Rabbit serum was used for blocking non-specific binding. The slides were then stained with primary antibody overnight at 4°C and secondary antibodies were incubated for 1 h at room temperature. Diaminobenzidine was applied before being counterstained with hematoxylin. Finally, the samples were sealed, viewed, and photographed by light microscope. The intensity of positive staining of ANK1, GRN and SEMA6A in glioma and non-tumor brain tissue sections were measured through Image-Proplus 6.0 software. All the images were taken using the same microscope and camera sets. The intensity of positive staining in tissue sections was analyzed by average optic density per stained area (μm^2^) (IOD/Area) for positive staining. Primers and Antibodies can be found in **Tables S2**, **S3**, respectively.

### Western blot

Protein was extracted from the human GBM cell line, U87. Total proteins were extracted in RIPA Lysis Buffer (Beyotime, China) with protease inhibitor cocktail (Beyotime, China). BCA assays (Beyotime, China) were utilized to quantify all proteins. 20 μg protein samples were separated onto 10% SDS-PAGE, transferred to PVDF membrane (Millipore, France) and revealed with ECL (EpiZyme, China). The blots were incubated with primary antibodies against ANK1 (Cloud-Clone Corp, USA), GRN (ABclonal, China), SEMA6A (CUSABIO, China), and GAPDH (ABclonal, China). The secondary antibodies used were HRP-conjugated anti-rabbit (ABclonal, China) antibodies.

### Cell transfection assays

Control siRNA, CRNDE siRNA and GRN siRNA were purchased from Genecreate Company (Genecreate, China). The sequences of siRNAs for the indicated target genes can be found in **Table S4**. ANK1 pcDNA3.1 vector (ANK1), SEMA6A pcDNA3.1 vector (SEMA6A) and empty vector (Vector) were subcloned into the vector pcDNA3.1 (Genecreate, China). U87 cells were transfected using lipofectamine 2000 (ThermoFisher, USA) according to the manufacturer’s protocols. qRT-PCR and western blot were performed at 48–72h later to assess the transfection efficiency.

### CCK8 assay and transwell assay

Cell Couting Kit-8 (CCK8, Biosharp, China) was utilized to perform CCK8 assay. Cells to be examined were seeded into 96-well plates with 4000 cells per well. 10μL CCK8 reagents were added into the wells. The whole process needs to avoid light. Then, these 96-well plates were incubated at 37°C and 5% CO_2_ for 2 h. Finally, the optical densities of the wells were read on a microplate reader at 450 nm. The experiments were performed in triplicate.

Transwell migration and invasion assays were carried out with transwell chambers (8.0μm pore size), which were pre-coated with (for invasion assay) or without (for migration assay) 50 μL matrigel (Conring, USA). Briefly, transwell chambers were placed into the 24-well culture plate, the chamber was called the upper chamber, and the culture plate was called the lower chamber. 10^^^4 cells were resuspended in 100 μL serum-free medium and seeded into the upper chamber, and 700 μL complete-medium supplemented with 20% FBS was added into the lower chamber. Then, these 24-well plates were incubated at 37°C and 5% CO_2_ for 48 h. Afterwards, cells were fixed with 4% paraformaldehyde for 20 min and stained with 0.1% crystal violet for 30 min. Five regions were randomly selected in each chamber to count migrating or invading cells. Photographs were taken in a light microscope and ImageJ Software was used for cell counts. The experiments were performed in triplicate.

## Results

### Constructing a background interaction network based on Reactome database

**Figure 1:**
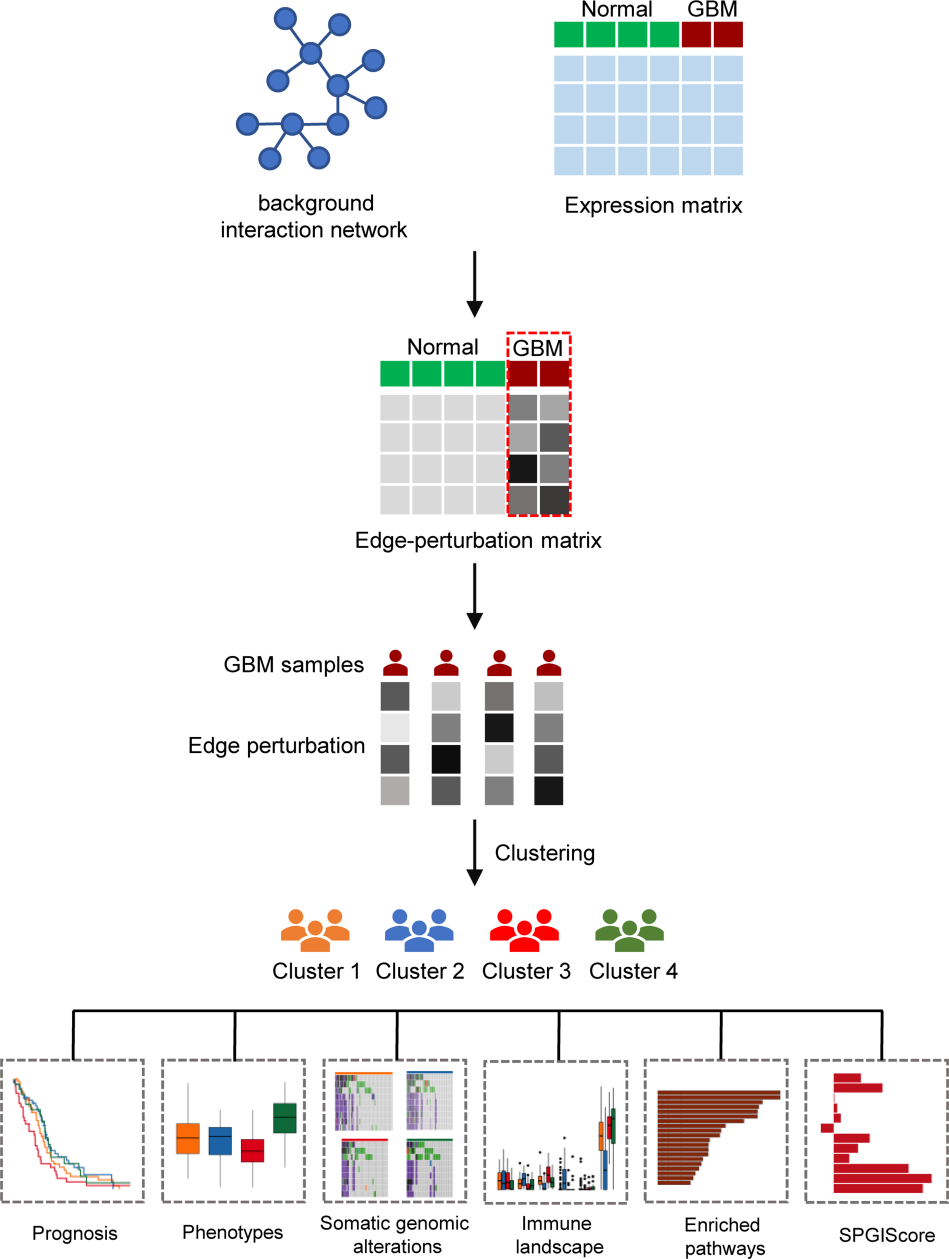
The flow diagram of the research process.

**Figure 2:**
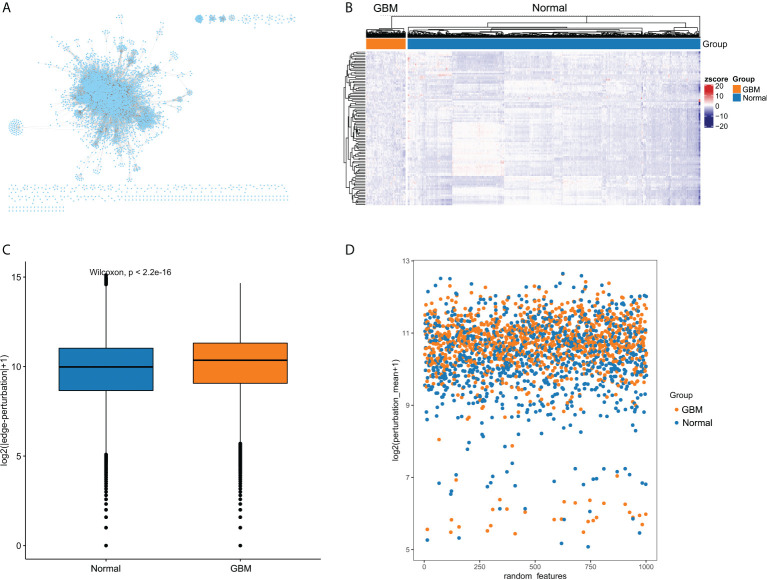
The edge-perturbation of gene interactions in GBM and normal samples. **(A)** The filtered background interaction network with 6194 nodes and 142974 edges. **(B)** The expression heatmap of nodes with top 100 degree in the background interaction network. **(C)** Comparing the edge-perturbation of 1000 randomly selected features between GBM and normal samples. **(D)** A scatterplot showed the edge-perturbation distribution of 1000 randomly selected features between GBM and normal samples.

The overall flow diagram of this study was presented in **Figure 1**. Based on molecular interactions from Reactome database, we constructed an original background interaction network, comprising a total of 7360 nodes and 169710 edges. Additionally, genes with zero expression over 70% samples in TCGA cohort or GTEx cohort were excluded. After correcting the plate batch effects, the GBM expression profiles contained 23890 genes and 155 samples, and the normal expression profiles contained 23890 genes and 1152 samples. Then, we filtered out 1166 nodes that were outside the 23890 genes and constructed a new background interaction network, which was composed of 6194 nodes and 142974 edges (**Figure 2A**). Obviously, the background interaction network was closely connected internally and most nodes had higher degrees. According to the degree of nodes in the background interaction network from large to small, the top 100 were selected and visualized as a heatmap (**Figure 2B**). In addition, the degree distribution of the background interaction network was illustrated in **Figure S1**, and the determination coefficient R^2^ was 0.674, indicating that this network was relatively scale free.

### Stronger edge perturbation in GBM samples

The perturbation of background interaction network inevitably leads to the change of interaction. The perturbation of gene pairs in the network can be reasonably used to reveal the pathological environment of individuals in disease states. To measure the perturbation degree of background interaction network at an individual level, we constructed EPMs for GBM and normal samples, respectively, based on their differences in gene expression fluctuations. To present the difference in edge-perturbation distribution between GBM and normal samples, we then randomly selected 1000 features from all gene interaction features. The edge-perturbation amplitudes of these 1000 features were quantified as log_2_(|△es|+1), and were significantly different between GBM and normal samples (**Figure 2C**). In addition, in order to visually present the difference in the edge-perturbation distribution between GBM and normal samples, the mean edge-perturbation amplitude of the 1000 selected features with a similar log_2_ transformation was plotted in **Figure 2D**. The edge perturbation of GBM samples (orange points) is stronger than that of normal samples (blue points). These findings provided a reliable basis for further analysis using EPM to explore the heterogeneity of GBM samples.

### Identification of four clusters of GBM samples based on the edge-perturbation matrix

**Figure 3:**
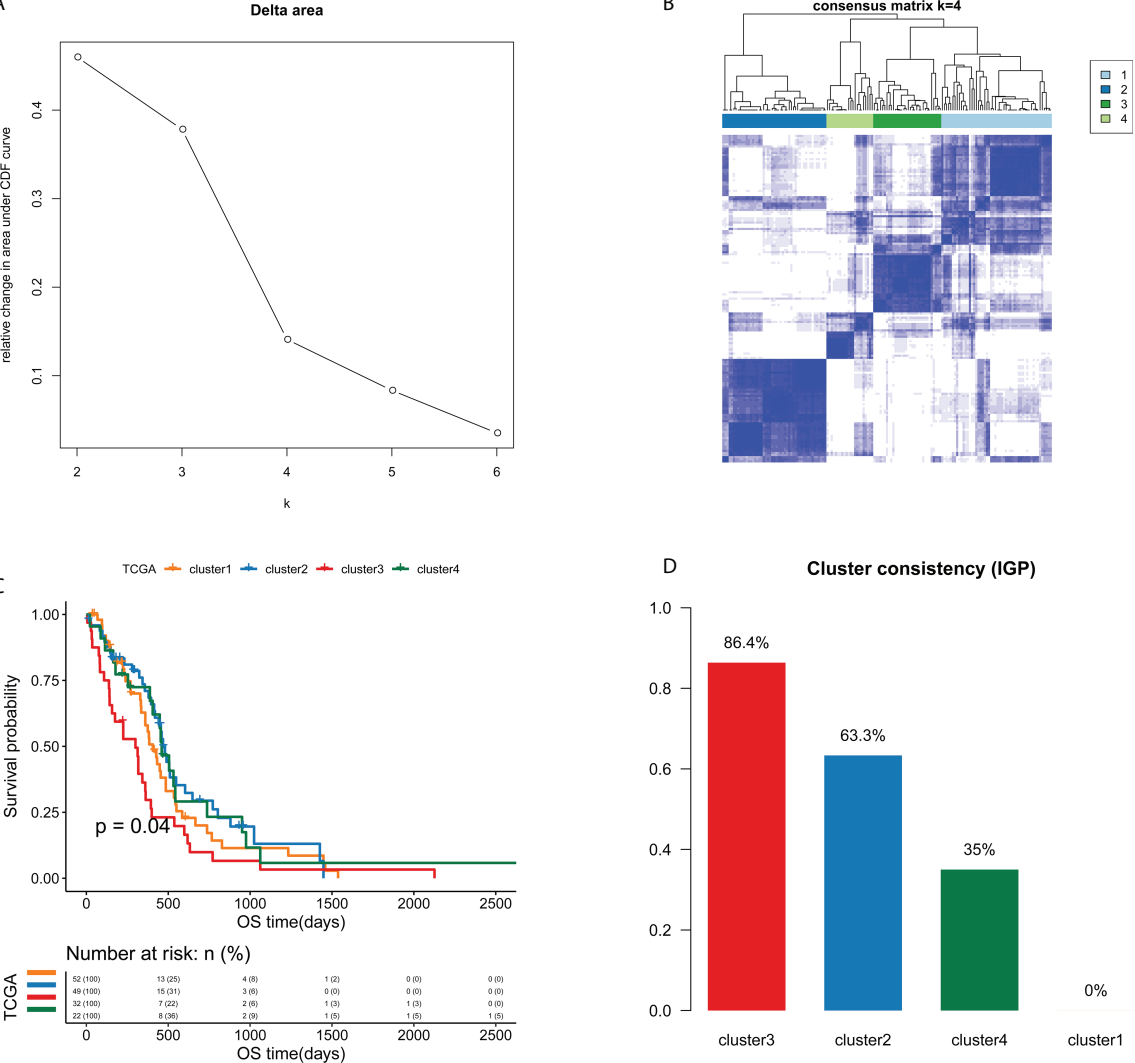
Unsupervised consensus clustering analysis identified four edge perturbation-based clusters of GBM samples in TCGA cohort. **(A)** Relative change in area under CDF curve for k=2-6. **(B)** Consensus clustering matrix for the optimal cluster number (k=4). **(C)** Kaplan-Meier curve showed the significant survival differences among edge perturbation-based clusters. **(D)** The IGP values of four edge perturbation-based clusters were calculated in CGGA-mRNA_array_301 cohort to evaluate the consistency between two cohorts.

A total of 21754 feature edges were selected from the EPM of GBM samples, and formed a network with 4402 genes (**Figure S2**). This network was also a scale-free network, whose determination coefficients R^2^ was 0.933 (**Figure S3**). Next, the clustering analysis of GBM samples was carried out based on 21754 selected feature edges. According to the relative change in the area under the CDF curve and the consensus heatmap, k = 4 was picked as the most optimal number of clusters (**Figures 3A, B** and **Figure S4**). As a result, 155 GBM samples from TCGA cohort were categorized into 4 clusters, namely cluster 1 (n=52), cluster 2 (n=49), cluster 3 (n=32) and cluster 4 (n=22). As the Kaplan-Meier curve showed, there were significant survival differences among four clusters (*P*=0.04, **Figure 3C**). Cluster 3 had the worst prognosis compared with other three clusters, while cluster 2 and cluster 4 exhibited relatively better prognosis.

To verify the clustering performance of edge perturbation, we also used another independent cohort of GBM samples (CGGA-mRNA-array_301) as the validation cohort. The IGP values of each cluster were calculated to evaluate the consistency within clusters. Results showed that there were relatively high IGP values of cluster 2 (63.3%) and cluster 3 (86.4%), suggesting that the sample clustering trend of the verification cohort may be more consistent with cluster 2 and cluster 3 (**Figure 3D**).

### Correlation of edge perturbation-based clusters with clinicopathological characteristics and somatic mutations

**Figure 4:**
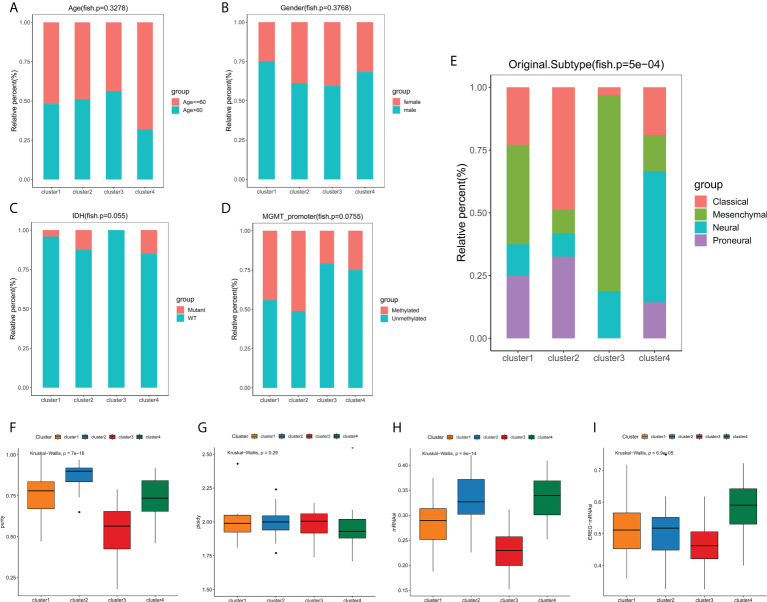
Phenotype heterogeneities among edge perturbation-based clusters. **(A–E)** The distributions of age **(A)**, gender **(B)**, IDH mutant status **(C)**, MGMT promoter methylation status **(D)** and transcriptomic subtypes **(E)** among edge perturbation-based clusters. **(F–I)** Comparison of tumor purity **(F)**, genome ploidy **(G)**, mRNAsi **(H)** and EREG-mRNAsi **(I)** among edge perturbation-based clusters.

We next explored the correlation between the clusters and clinicopathological phenotypes. Fisher exact tests showed that there were no significant differences in terms of age, gender, IDH mutation status and MGMT promoter methylation status among edge perturbation-based clusters (**Figures 4A–D**). However, the distribution of transcriptomic subtypes differed significantly (**Figure 4E**). Cluster 2 was mainly comprised of Classical and Proneural subtypes. Mesenchymal subtype and Neural subtype occupied a dominant portion of Cluster 3 and Cluster 4, respectively. Further, we sought to assess the genomic heterogeneity indicators among four clusters. Cluster 3, with the worst prognosis, had a significantly lower tumor purity than the other three clusters (**Figure 4F**). No significant differences in genome ploidy were detected among four clusters (**Figure 4G**). In previous studies, the mRNAsi was used for assessing the degree of oncogenic dedifferentiation. Here, we obtained the mRNAsi of GBM samples at transcript level and epigenetically regulated level, and found that cluster 3 had lower mRNAsi and EREG-mRNAsi (**Figures 4H, I**).

**Figure 5:**
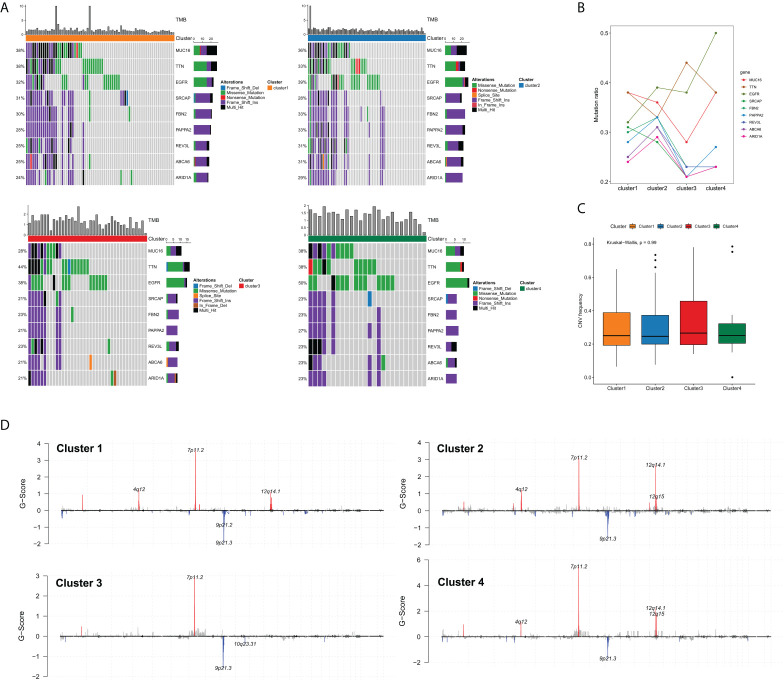
Comprehensive analyses of genomic alterations among edge perturbation-based clusters. **(A)** The somatic mutation profiles showed the mutation distribution of nine common genes with mutation frequencies in the top50 in all edge perturbation-based clusters. **(B)** A line chart presented the difference in the mutation ratios of nine genes among edge perturbation-based clusters. **(C)** Comparison of CNV frequency among edge perturbation-based clusters. **(D)** Copy number profiles for edge perturbation-based clusters showed gains and losses of copy numbers of genes, which were placed based on their location on chromosomes, ranging from chromosome 1 to chromosome 22.

Previous studies have demonstrated that the somatic mutations of key oncogenes or tumor-suppressor genes were tightly correlated with the survival and therapeutic response of cancer patients (20). Accordingly, we were particularly interested in whether the edge perturbation-based clusters differed in somatic mutations. **Figure 5A** showed the mutation distribution of nine common genes with mutation frequencies in the top50 in four clusters. In order to characterize the mutation proportion of high-frequency mutated genes more granularly, we drew a line chart of these nine genes, and easily observed that there were noticeable differences among four clusters (**Figure 5B**). Particularly, Cluster 4 had the highest mutation rate of epidermal growth factor receptor (EGFR). It has been demonstrated that EGFR is one of the most frequently altered genes in GBM, and the most common mutation type is the in-frame deletion of exons 2-7 (EGFRvIII), which can reduce the apoptosis and increase proliferation and invasiveness of GBM cells (21–23). Cluster 3 had the highest mutation rate of TTN, whose mutation was found to be correlated with increased TMB and greater response to ICI therapy in patients with solid tumors (24). However, the mutation ratios of MUC16, SRCAP, FBN2, PAPPA2, REV3L, ABCA6 and ARID1A were all lowest in cluster 3 compared with the other three clusters. The frequency of CNV was also compared, whereas no significant difference was found among edge perturbation-based clusters (**Figure 5C**). The distributions of CNV regions were visualized in **Figure 5D**.

### Different tumor immune landscapes and immunotherapeutic responses among edge perturbation-based clusters

**Figure 6:**
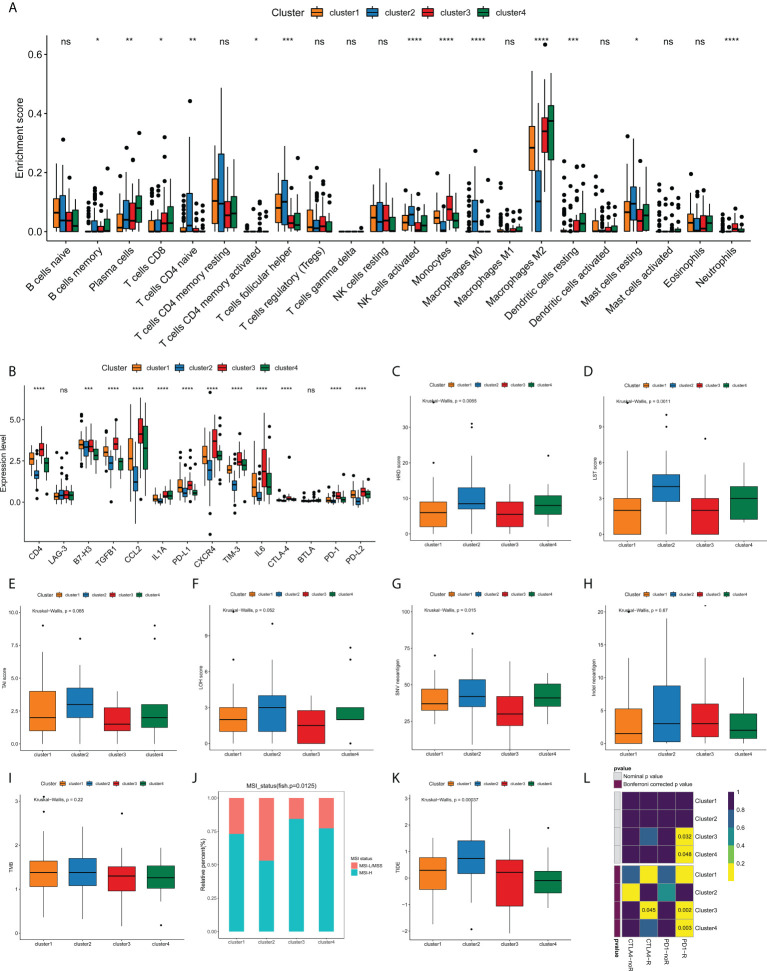
Analyses of tumor immune landscape and prediction of immunotherapeutic responses for edge perturbation-based clusters. **(A)** The infiltration levels of 22 immune cells in different edge perturbation-based clusters. **(B)** The expression levels of immune checkpoints, cytokines, and receptors in different edge perturbation-based clusters. **(C–H)** Comparison of potential indicators reflecting the immunogenicity of edge perturbation-based clusters, including HRD score **(C)**, LST score **(D)**, TAI score **(E)**, LOH score **(F)**, SNV neoantigen load **(G)**, and Indel neoantigen load **(H)**. **(I, J)** Comparison of TMB **(I)** and distribution of MSI status **(J)** among edge perturbation-based clusters. MSI-L, MSI-Low; MSS, microsatellite stability; MSI-H, MSI-High. **(K, L)** Prediction of responses to immune checkpoint inhibitor (ICI) therapy for edge perturbation-based clusters through TIDE algorithm **(K)** and Subclass mapping analysis **(L)**. *p < 0.05, **p < 0.01, ***p < 0.001, ****p < 0.0001, and ns, No significance.

Currently, the clinical efficacy of immunotherapy in GBM patients is far from satisfactory. Some patients have seen notable response with immunotherapeutic intervention, whereas a substantial proportion of patients have experienced small or no clinical benefit with the same treatment (25, 26). The heterogeneity of immune landscape is a major factor complicating therapeutic options, as well as leading to different outcomes. Thus, we sought to investigate the difference in tumor immune landscape among edge perturbation-based clusters. Immune cells are the crucial component of tumor microenvironment. By using CIBERSORT algorithm, we found that there were prominent differences in the distribution of most immune cells among four clusters (**Figure 6A** and **Figure S5**). The expression levels of most immune checkpoints, cytokines and receptors also varied substantially across clusters (**Figure 6B**). Especially, Cluster 2 was remarkably rich in innate immune cells, including naïve CD4^+^ T cells, follicular helper T cells, activated NK cells, M0 macrophages and resting mast cells, but exhibited lower infiltration of M2 macrophages, which is the main immunosuppressive cell in immune microenvironment of GBM. In addition, Cluster 2 had significantly lower expression levels of immune checkpoints, such as PD-1, PD-L1, PD-L2, CTLA-4, LAG-3, TIM-3 and B7H3, and immunosuppressive cytokines or receptors, including CCL2, CXCR4, IL1A and IL6. These may suggest more active immune response and antitumor reaction in Cluster 2, which also matched a survival advantage of Cluster 2. Conversely, an evident immunosuppressive landscape was observed in Cluster 3 with low infiltrations of innate immune cells, high infiltration of M2 macrophages, and high expression levels of immune checkpoints, immunosuppressive cytokines, and receptors. It could be reasoned that Cluster 3 was in a stronger immunosuppressive microenvironment, which contributed to tumor immune escape and dismal survival.

In addition to immunosuppressive microenvironment, inherent immune escape, which means that tumor cells can mediate their own immune escape directly, also plays an essential role in the process of escaping immune-mediated killing. Tumor cell immunogenicity is well known as an important aspect of inherent immune escape. Next, some potential indicators were used to reflect the level of tumor cell immunogenicity, including HRD score, LST score, LOH score, SNV neoantigen load, Indel neoantigen load. As **Figures 6C–H** illustrated, all HRD score, LST score and SNV neoantigen load differed significantly among four clusters. Particularly, Cluster 2 showed higher levels of HRD score, LST score and SNV neoantigen load compared with clusters, whereas Cluster 3 exhibited the opposite trend. These results demonstrated that Cluster 3 might have a stronger inherent immune escape ability.

Currently, immune checkpoint inhibitor (ICI) therapy has undoubtedly been a very promising strategy of immunotherapy, which made a breakthrough in antitumor treatment. First, we compared the TMB and the distribution of MSI status among edge perturbation-based clusters (**Figures 6I, J**). No apparent difference in TMB was observed. Interestingly, Cluster 3 and Cluster 4 had a greater proportion of MSI-H status, which has been validated as a positive indicator of stronger response to ICI therapy (27). Consistent result was obtained by TIDE algorithm. Cluster 3 and Cluster 4 exhibited lower TIDE scores, which also represented a stronger response to ICI therapy (**Figure 6K**). Further, subclass mapping analysis revealed that Cluster 3 was more likely to respond to PD-1 inhibitor (Bonferroni corrected *P* = 0.002) and CTLA-4 inhibitor (Bonferroni corrected *P* = 0.045), and Cluster 4 showed response to CTLA-4 inhibitor (Bonferroni corrected *P* = 0.003; **Figure 6L**). These interesting findings suggested that the edge perturbation-based clusters may be informative for immunotherapeutic options to eliminate GBM.

### Distinct pathway enrichments of edge perturbation-based clusters

**Figure 7:**
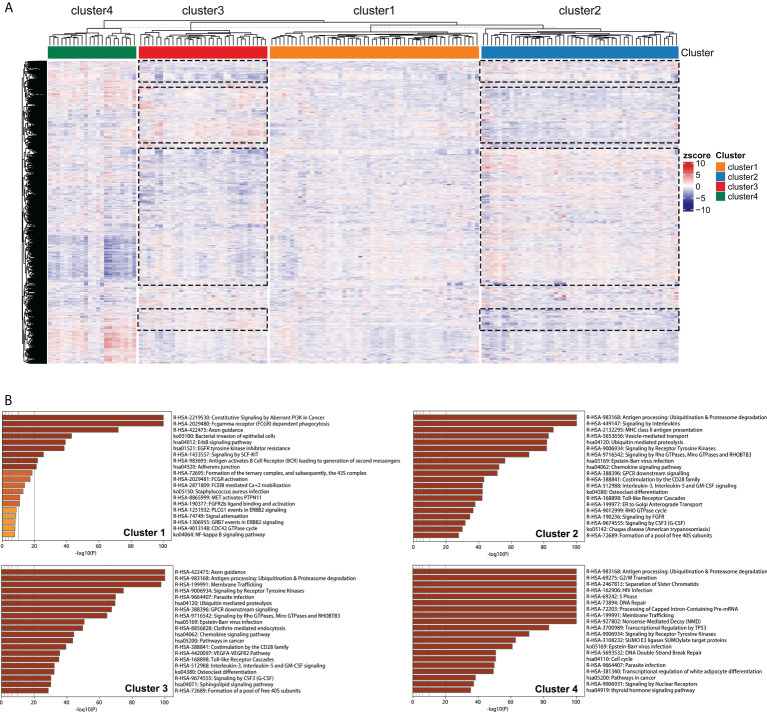
Pathway enrichment analyses of edge perturbation-based clusters. **(A)** Clustering of the 21754 feature edges of the GBM sample matrix. The dotted boxes represent the identified blocks with opposite trend between Cluster 2 and Cluster 3. **(B)** The specific pathway enrichments of edge perturbation-based clusters.

Clustering of 21754 feature edges of the GBM sample matrix was visualized as a heatmap (**Figure 7A**). The distribution of color bars was not consistent in specific blocks at the same level, representing that the perturbation patterns of specific feature edges differed among edge perturbation-based clusters. The blue color corresponds to a negative perturbation, and the red color corresponds to a positive perturbation. Remarkably, most blocks were in the opposite perturbation directions between Cluster 2 and Cluster 3. This revealed that the disorder mechanisms of Cluster 2 and Cluster 3 were in close agreement but in different directions. This finding was also in corroboration with the previous results that the performances of these two clusters were opposite in multiple aspects.

**Figure 7B** showed the specific pathway enrichments of each edge perturbation-based cluster. The pathway with the highest enrichment in Cluster 1 was constitutive signaling by aberrant PI3K in cancer, which is in the central position of the signaling cascade affecting GBM progression (28). The enriched pathways of Cluster 2 and Cluster 3 were highly overlapped, including some immune-associated pathways, such as antigen processing: ubiquitination and proteasome degradation, chemokine signaling pathway, and interleukin-3, interleukin-5 and GM-CSF signaling. This was in correspondence with our speculation that the disorder mechanisms of Cluster 2 and Cluster 3 were similar but in different directions. The enriched pathways of Cluster 4 were mainly correlated with cell cycle and genetic information processing, such as G2/M transition, DNA repair, processing of capped intron-containing pre-mRNA, transcriptional regulation by TP53, DNA double-strand break repair and cell cycle.

### Constructing and verifying the sample-specific perturbation of gene interaction score

**Figure 8:**
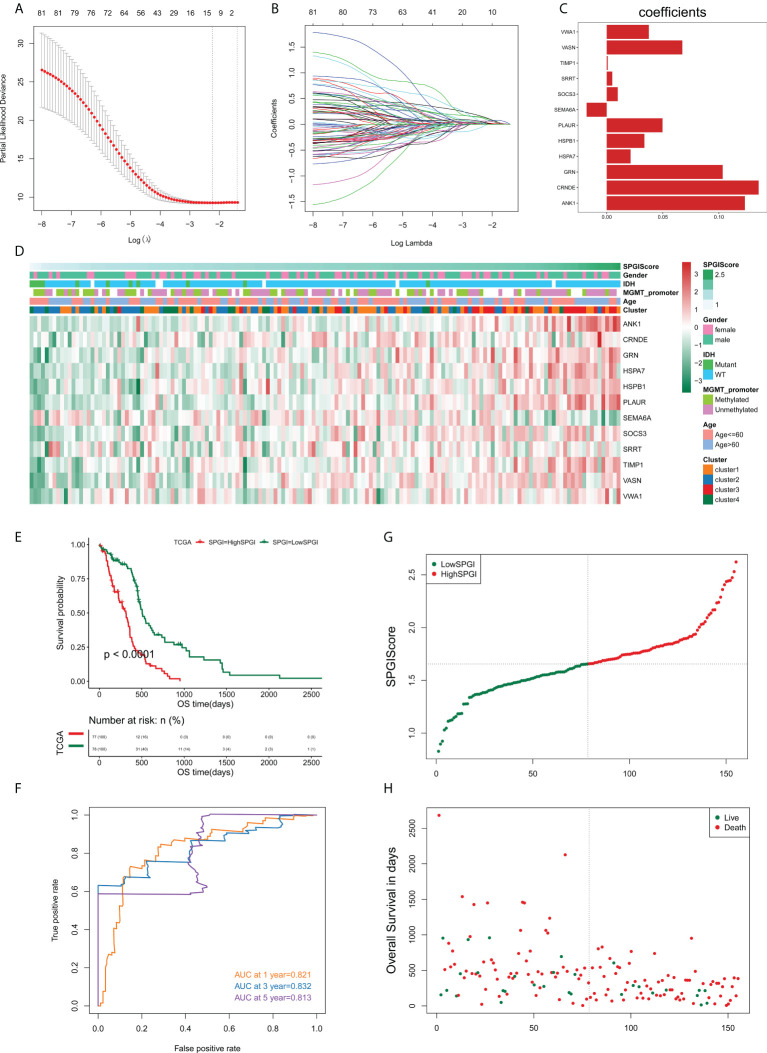
Construction of the sample-specific perturbation of gene interaction score (SPGIScore) in TCGA cohort. **(A, B)** The least absolute shrinkage and selection operator (LASSO) regression was performed with the minimum criteria. **(C)** LASSO coefficients of 12 hub differentially expressed genes. **(D)** A heatmap showed the correlation between SPGIScore and clinicopathological features, edge perturbation-based clusters, and expression levels of 12 hub DEGs, respectively. **(E)** Kaplan-Meier curve of high- and low-SPGIScore subgroups. **(F)** ROC curve analysis of SPGIScore in predicting 1-, 3- and 5-year OS. **(G, H)** The distribution plot of SPGIScore and survival status.

We sought to construct a SPGIScore with prognostic value. First, the DEGs were screened out by pairwise comparison among four edge perturbation-based clusters. The upset plot showed the number of DEGs of each pairwise group and the number of intersections of different combinations (**Figure S6**). A total of 1058 DEGs were selected as they were identified at least in four pairwise comparisons. This was done in order to avoid the selected DEGs that only differed from one cluster with the other three clusters, respectively. By performing univariate Cox regression analyses with the criterion of *P*< 0.05, eighty-one prognostic DEGs were identified from 1058 DEGs. The forest plot presented the top 20 prognostic DEGs according to the *P* value from small to large (**Figure S7**). The LASSO regression was then performed based on these eighty-one prognostic DEGs, twelve of which stood out for the construction of SPGIScore (**Figures 8A** **–C**). As shown in **Figure 8D**, SPGIScore was ranked from low to high to show the correlation between SPGIScore and clinicopathological features, edge perturbation-based clusters, and expression levels of twelve hub DEGs, respectively. Next, GBM patients were stratified into the high- and low-SPGI groups using the median SPGIScore as the cut-off value. The Kaplan-Meier curve suggested that patients in high-SPGI group exhibited worse survival outcomes in TCGA cohort (**Figure 8E**), CGGA-mRNAseq_325 cohort (**Figure S8A**) and Rembrandt cohort (**Figure S8E**). A satisfactory prognosis predictive power of SPGIScore was confirmed by the area under the receiver operating characteristic (ROC) curve (AUC) for 1-, 3- and 5-year overall survival, which were 0.821, 0.832 and 0.813 in the TCGA cohort (**Figure 8F**), 0.591, 0.678 and 0.751 in the CGGA-mRNAseq_325 cohort (**Figure S8B**), 0.568, 0.661 and 0.673 in the Rembrandt cohort (**Figure S8F**). The distribution plot of SPGIScore and survival status showed that the higher the risk score, the more deaths of GBM patients (**Figures 8G, H** and **Figures S8C, D, G, H**).

### Expression levels and biological functions of selected SPGIScore-related genes in GBM

**Figure 9:**
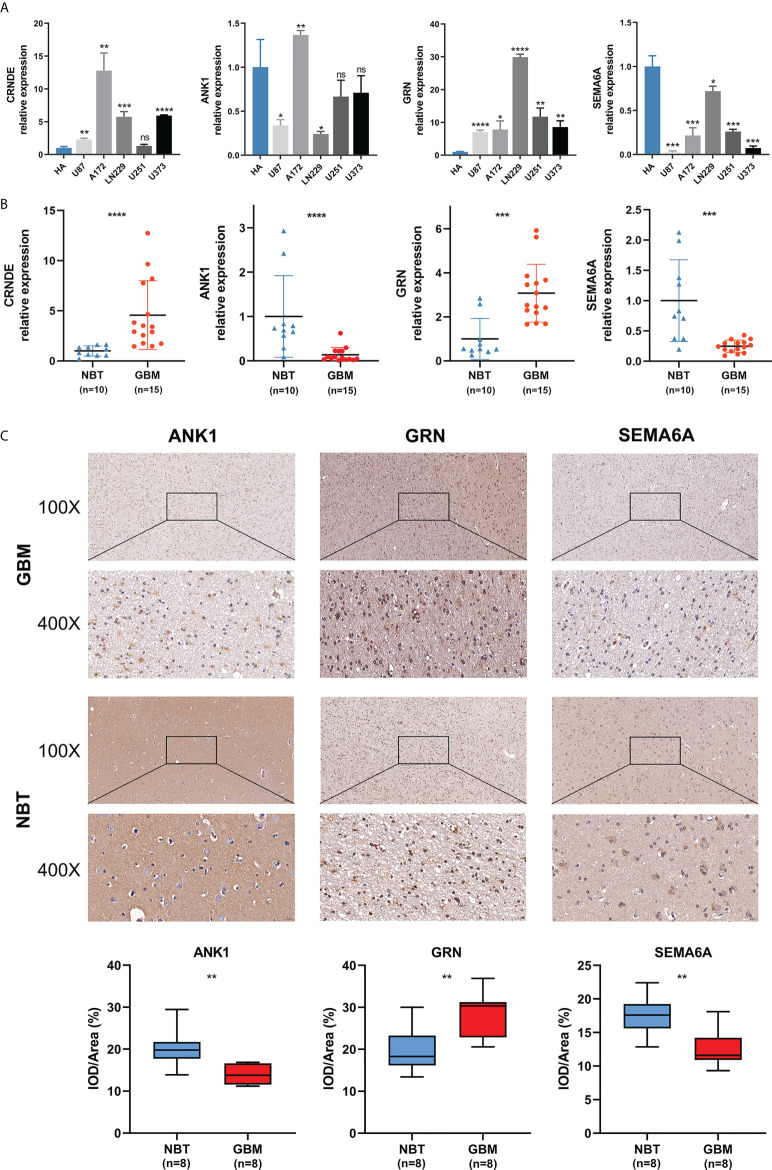
Validation of the expression levels of selected SPGIScore-based genes. **(A)** Scatter plots of differential transcript levels between CRNDE, ANK1, GRN and SEMA6A in GBM cell lines and normal human astrocytes cell lines (HA). **(B)** Scatter plots of differential transcript levels between CRNDE, ANK1, GRN and SEMA6A in GBM and NBT. **(C)** Representative IHC staining images. GBM, glioblastoma; NBT, non-tumor brain tissues; IOD/Area Integrated optical density per stained area. *p < 0.05, **p < 0.01, ***p < 0.001, ****p < 0.0001, and ^ns^No significance.

We detected the expression levels of four selected SPGIScore-based genes (CRNDE, ANK1, GRN and SEMA6A) in cell lines and tissue samples. As **Figures 9A** **, B** showed, the transcript levels of CRNDE and GRN were both elevated in human GBM cell lines and GBM tissues, while the transcript levels of ANK1 and SEMA6A exhibited an overall downward trend in human GBM cell lines and GBM tissues. As CRNDE is a non-coding gene, only the protein levels of ANK1, GRN and SEMA6A were qualitatively assessed *via* IHC staining. It could be seen intuitively that compared with NBT, GRN was up-regulated, but ANK1 and SEMA6A were down-regulated in GBM tissues (**Figure 9C**).

**Figure 10:**
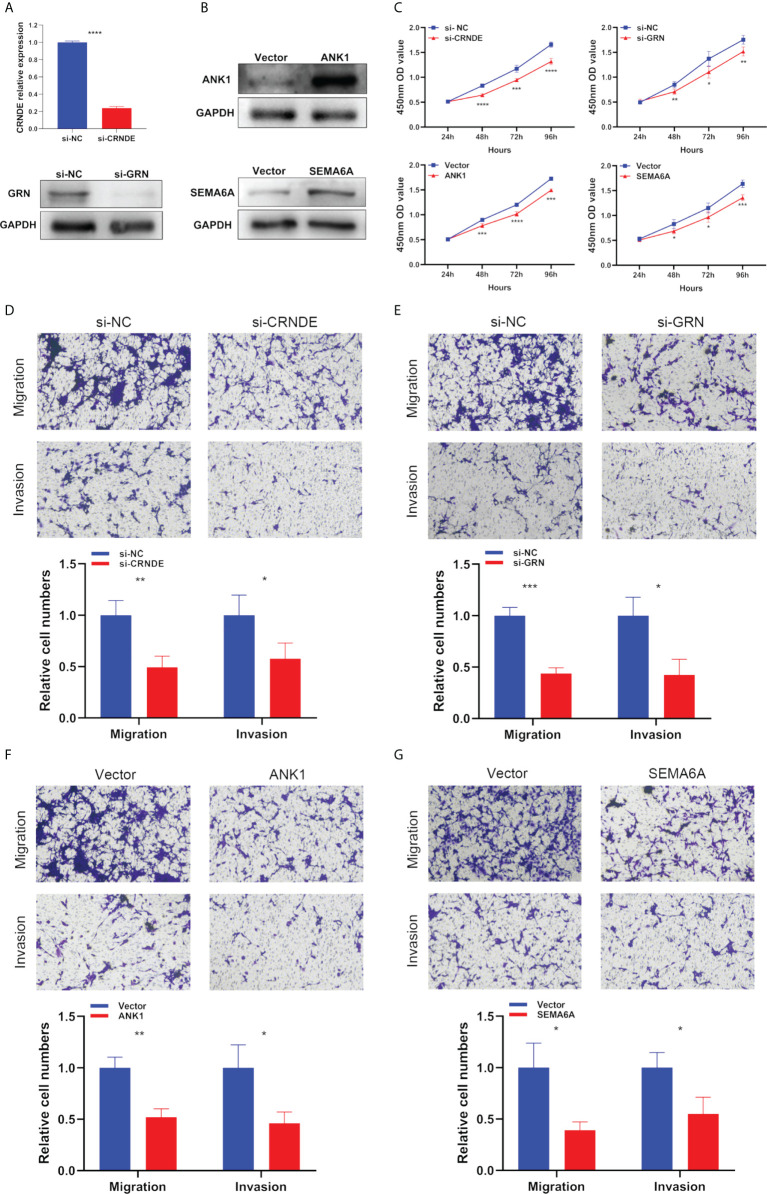
The biological functions of selected SPGIScore-based genes in GBM. **(A, B)** Verification of knockdown efficiency of CRNDE and GRN, and overexpression efficiency of ANK1 and SEMA6A in U87 cell line. **(C–G)** The biological functions of four selected SPGIScore-based genes on U87 cell line were verified by CCK-8 and Transwell assays. *p < 0.05, **p < 0.01, ***p < 0.001, and ****p < 0.0001.

Next, we used specific CRNDE-targeting and GRN-targeting siRNAs to knockdown the expression levels of CRNDE and GRN in U87 cells (**Figure 10A**). Meanwhile, we transiently transfected overexpression plasmid of ANK1 and SEMA6A into U87 cells, resulting in increased expression levels of ANK1 and SEMA6A, respectively (**Figure 10B**). Results of CCK-8 assay and Transwell assay showed that CRNDE and GRN knockdown suppressed the cell proliferation, migration and invasion of U87 cells, and ANK1 and SEMA6A overexpression also suppressed the cell proliferation, migration and invasion of U87 cells *in vitro* (**Figures 10C** **–G**).

## Discussion

Many studies have attempted to establish novel subtyping methods for better understanding of cancer heterogeneity. Most of these methods were based on gene expression, inevitably leading to the instability results due to the variability of gene expression profiles across time and condition. In this study, we applied a more stable and reliable method, sample-specific edge perturbation in the gene interaction network, to explore the heterogeneity of GBM. We identified four clusters of GBM samples. The heterogeneities among clusters were reflected in lots of aspects, including prognosis, phenotypic changes, somatic genomic alterations, immune landscapes, immunotherapeutic responses, and enriched pathways. We also constructed a SPGIScore based on the differential gene expression among four clusters. The SPGIScore was confirmed to possess a robust prognostic predictive ability. The expressions of genes involved in the SPGIScore were also validated at the cellular and tissue levels.

The application of network science to cancer genomics has opened new avenues for the discovery and molecular characterization of cancer subtypes (29). Developing network-based methods is promising for disclosing the nature of GBM heterogeneity. Previous investigators have done much exploration in this area. For instance, Xu et al. developed the weighted similarity network fusion (WSNF) method and identified three GBM subtypes with significantly different survival patterns and enriched pathways (30). Guo et al. exploited CSPRV (cancer subtype prediction using RV2), a method that incorporates multi-sources transcriptome expression data and heterogeneous biological networks, to successfully identify more clinically meaningful GBM subtypes (31). Notably, these network-based methods targeted the gene sets in a network as the main body and underestimated the weight of interactions among genes. The sample-specific edge perturbation method used in this study had an excellent utilization of gene interaction information. In brief, this method used the relative gene expression value to estimate the perturbation of gene interactions, which further represented the perturbation of interaction network (11). In our study, there was a striking difference in the perturbation of interaction network between GBM and normal samples, which confirmed that the perturbation of interaction network was able to reflect the individual health status. Moreover, variation among individuals in the perturbation degree of interaction network was also instructive for understanding the heterogeneity of GBM. Our study corroborated that the edge perturbation-based method reached a satisfactory discrimination power to multidimensional heterogeneities of GBM. It is especially commendable that the edge perturbation-based method exhibits potential value for predicting prognosis and immunotherapeutic response, which may shed new light on individualized diagnosis and therapies.

Undeniably, there is a long path ahead before the clinical application of the edge perturbation-based method. The good news is that the sequencing technology has shown a spurt of development, and has gradually gained popularity. A ready-to-use reprocessing tool for sequencing data will help clinicians quickly and accurately assess the individual edge perturbation in the gene interaction network. In addition, more related studies on other cancer types are urgently needed.

## Data availability statement

Publicly available datasets were analyzed in this study. This data can be found here: TCGA, CGGA, and GTEx.

## Author contributions

JZ, YQ, and ZW conceived and designed this study. JZ and ZW performed the data analysis and figure plotting. Experiments at cell and tissue levels were completed by JZ and ZW. JZ and YQ were involved in writing original draft. XW and XJ were responsible for the critical reading of the manuscript. All authors contributed to the article and approved the submitted version.

## Funding

This work was supported by National Natural Science Foundation of China (No.81974390).

## Acknowledgments

We all authors sincerely acknowledge the contributions from the TCGA, CGGA, and GTEx projects.

## Conflict of interest

The authors declare that the research was conducted in the absence of any commercial or financial relationships that could be construed as a potential conflict of interest.

## Publisher’s note

All claims expressed in this article are solely those of the authors and do not necessarily represent those of their affiliated organizations, or those of the publisher, the editors and the reviewers. Any product that may be evaluated in this article, or claim that may be made by its manufacturer, is not guaranteed or endorsed by the publisher.

## Abbreviations

GBM, Glioblastoma multiforme; TCGA, The Cancer Genome Atlas; SPGIScore, sample-specific perturbation of gene interaction score; GTEx, Genotype-Tissue Expression database; CGGA, Chinese Glioma Genome Atlas; GO, Gene Ontology; SD, standard deviation; IGP, in-group proportion; OCLR, one-class logistic regression; LST, large-scale transition; TAI, telomeric allelic imbalance; LOT, loss of heterozygosity; HRD, Homologous recombination deficiency; TIDE, Tumor Immune Dysfunction and Exclusion; ICI, immune checkpoint inhibitor; TMB, tumor mutation burden; mut/Mb, mutations per megabase; MSI, microsatellite instability; MSS, microsatellite stability; CNV, copy number variation; DEGs, differently expressed genes; LASSO, least absolute shrinkage and selection operator; NBT, non-tumor brain tissues; qRT-PCR, Quantitative real-time polymerase chain reaction; IHC, Immunohistochemistry; EGFR, epidermal growth factor receptor; ICI, immune checkpoint inhibitor; ROC, receiver operating characteristic; WSNF, weighted similarity network fusion; CSPRV, cancer subtype prediction using RV2.
