# Supplementary material for: Exploring the multidimensional heterogeneities of glioblastoma multiforme based on sample-specific edge perturbation in gene interaction network
Source: Front Immunol. 2022 Aug 29;13:944030. doi: 10.3389/fimmu.2022.944030 (PMC9464945; doi:10.3389/fimmu.2022.944030)
Supplement: Supplementary file 1 [file DataSheet_1.docx]

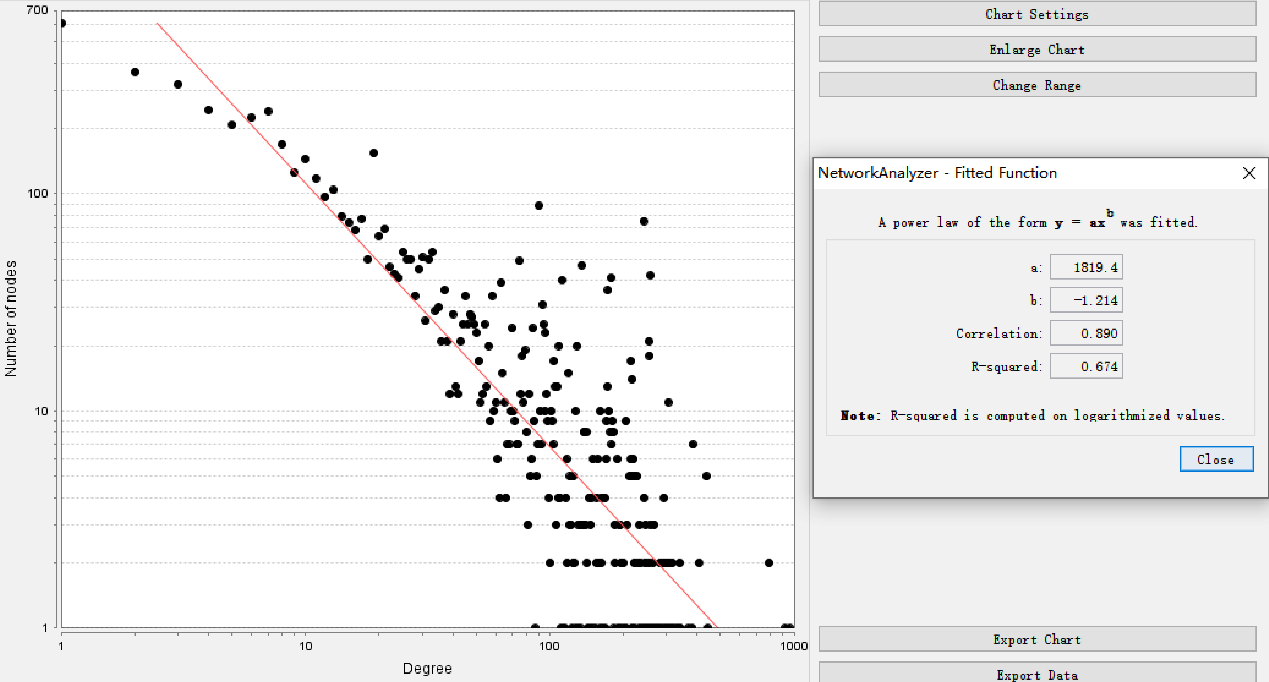
 **Fig. S1.** The nodes degree distribution and determination coefficients R^2^ of the background interaction network.


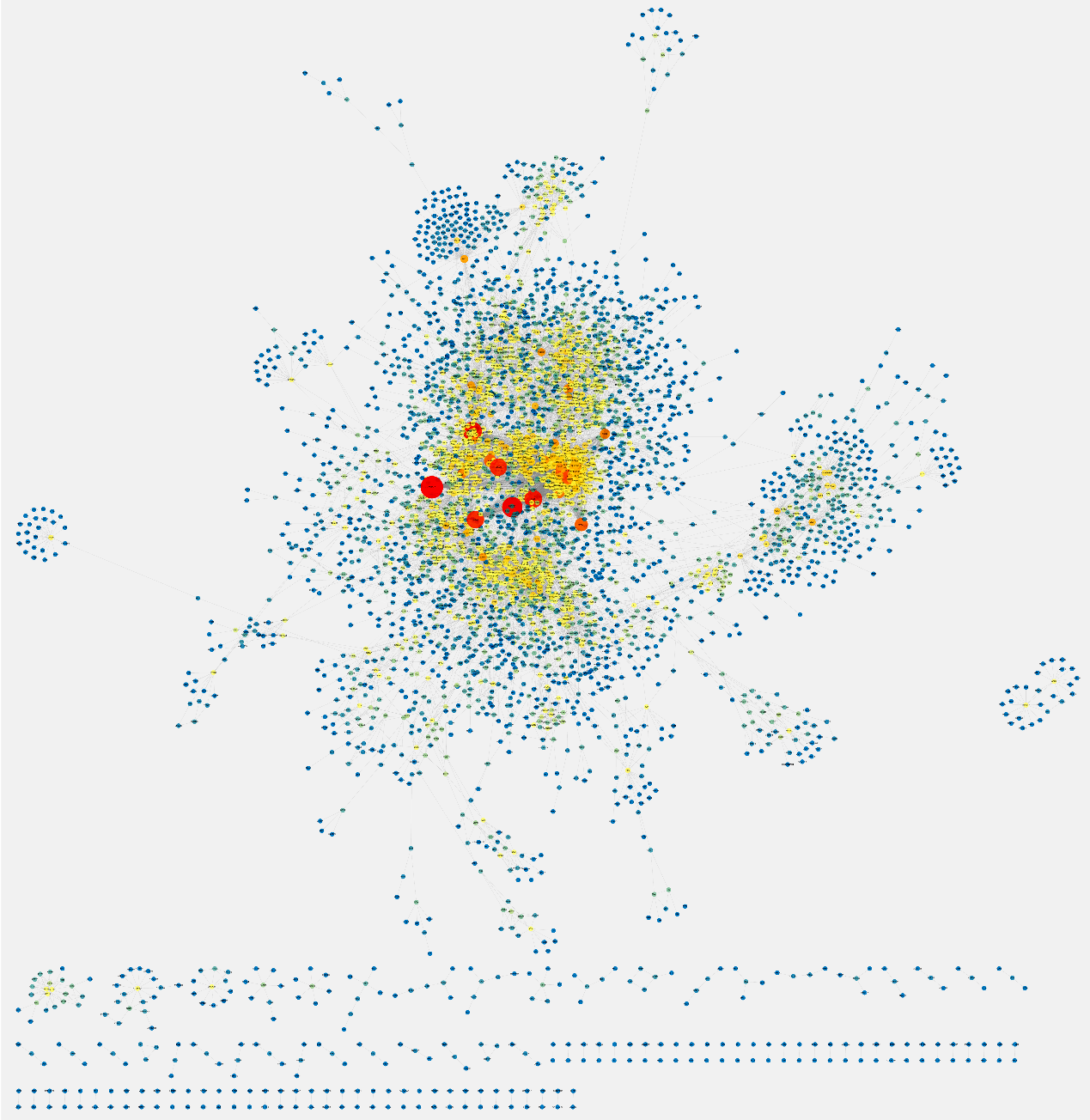


**Fig. S2.** The network of 21754 feature edges with 4402 genes selected from the edge-perturbation matrix of GBM samples. The different colors represent different gene degrees. Blue, green, yellow, orange, and red correspond to the minimum to maximum degrees.


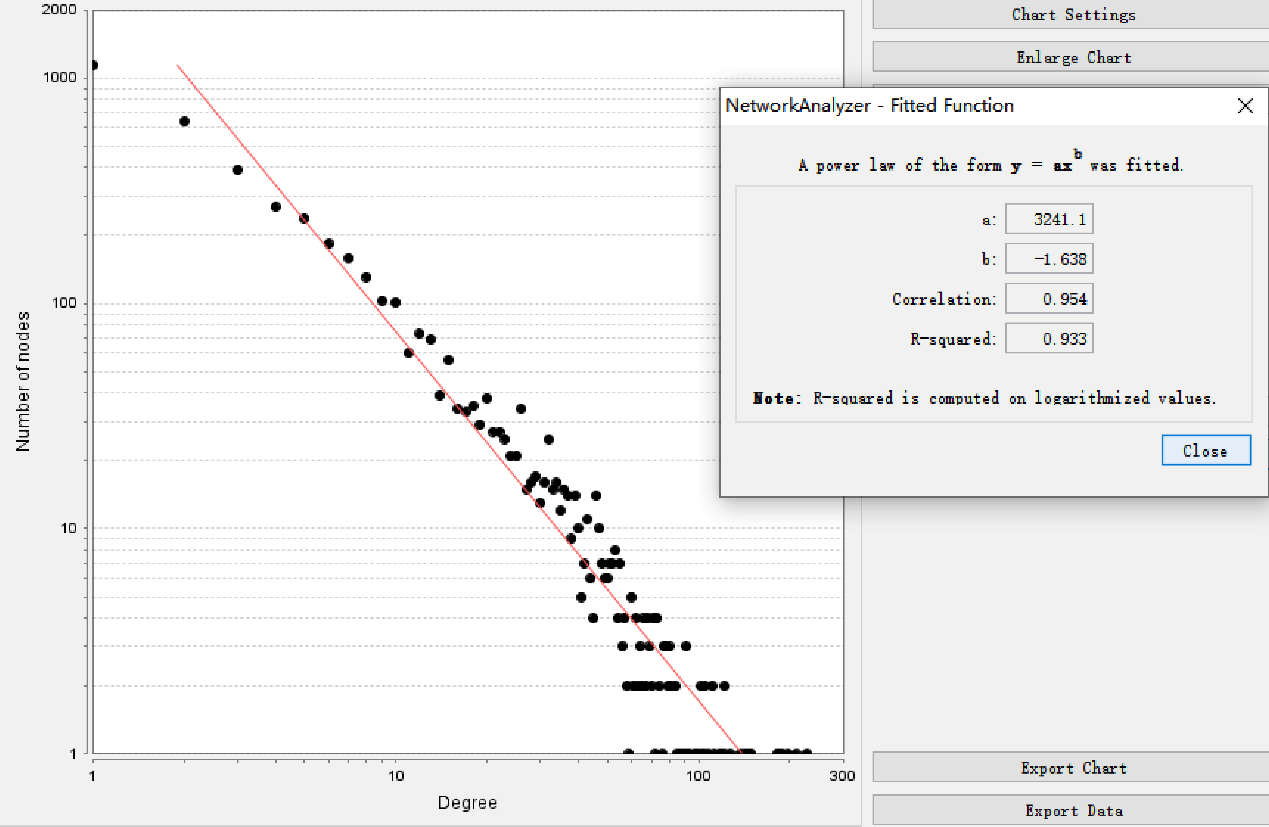


**Fig. S3.** The nodes degree distribution and determination coefficients R^2^ of the network comprising 21754 feature edges with 4402 genes selected from the edge-perturbation matrix of GBM samples.


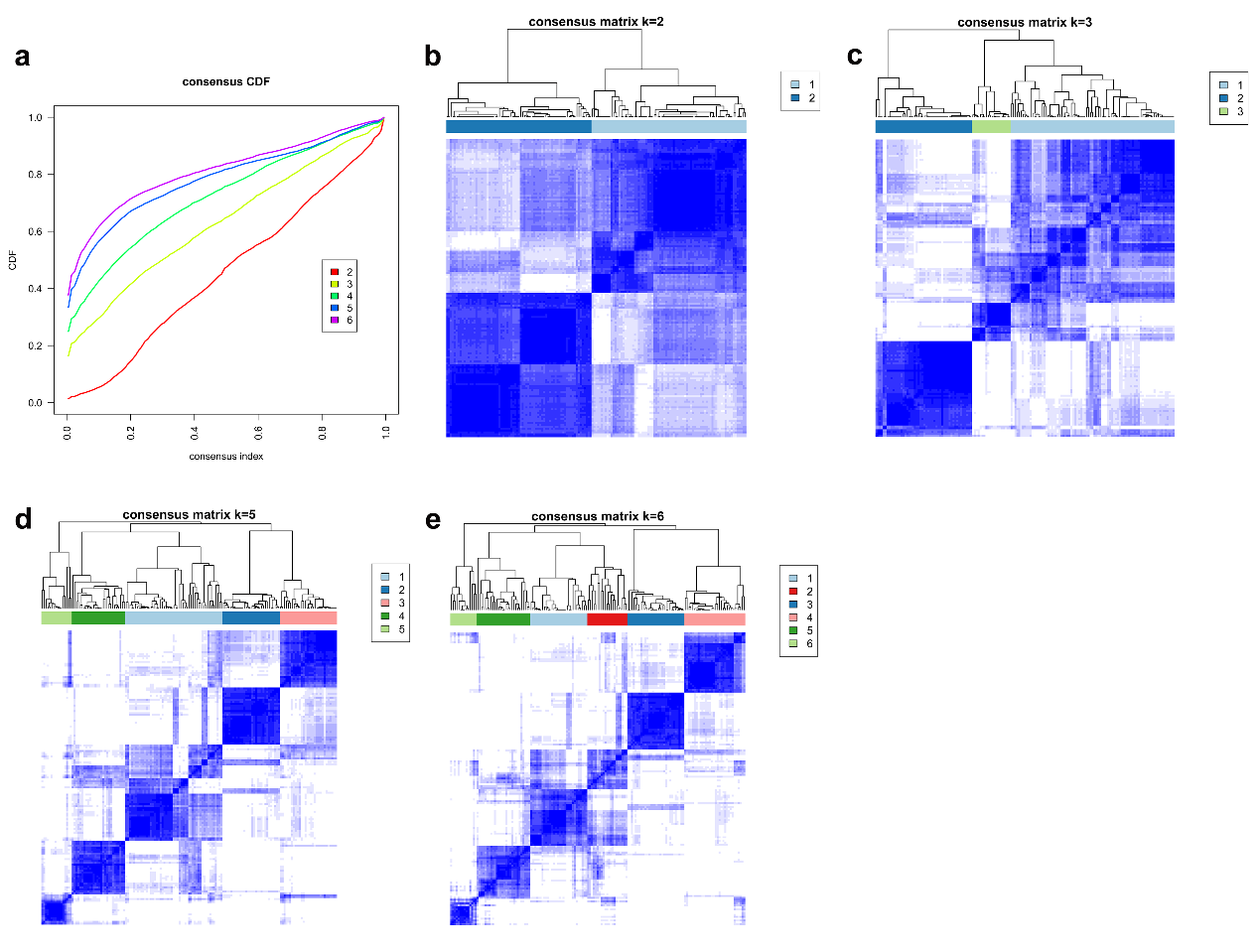


**Fig. S4.** Unsupervised consensus clustering analysis based on 21754 selected feature edges from the edge-perturbation matrix of GBM samples. (a) Consensus clustering CDF for k=2-6. (b) The consensus heatmaps for k=2, 3, 5 and 6.


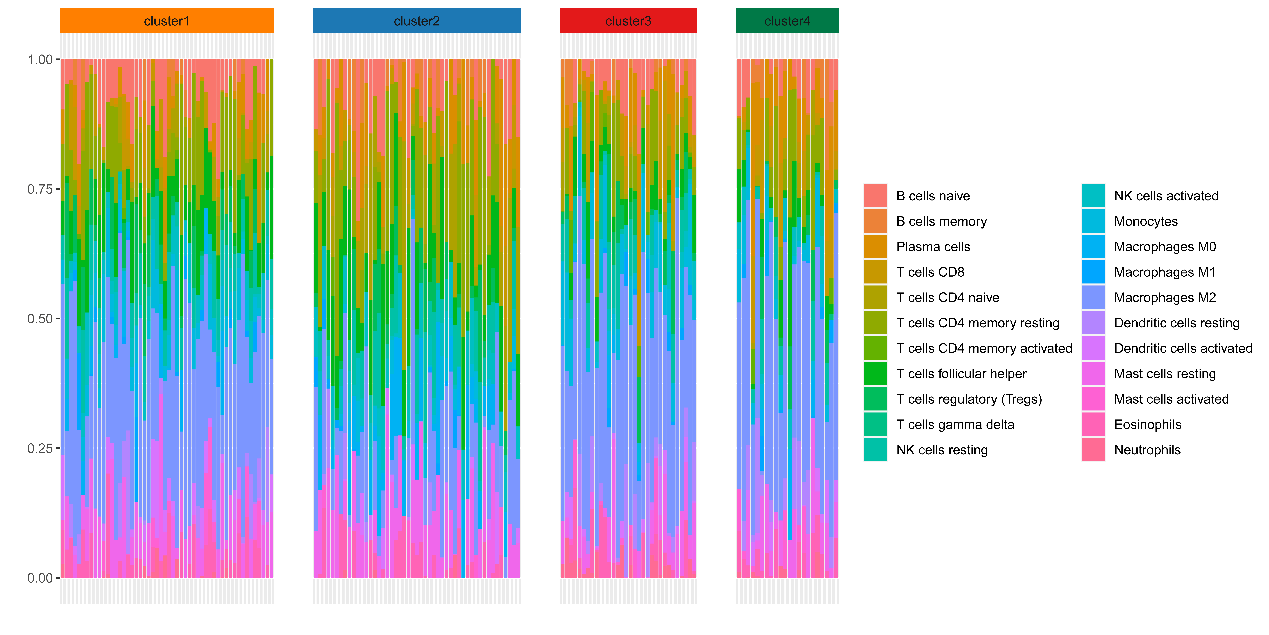


**Fig. S5.** The proportions of 22 immune cells for four edge perturbation-based clusters.


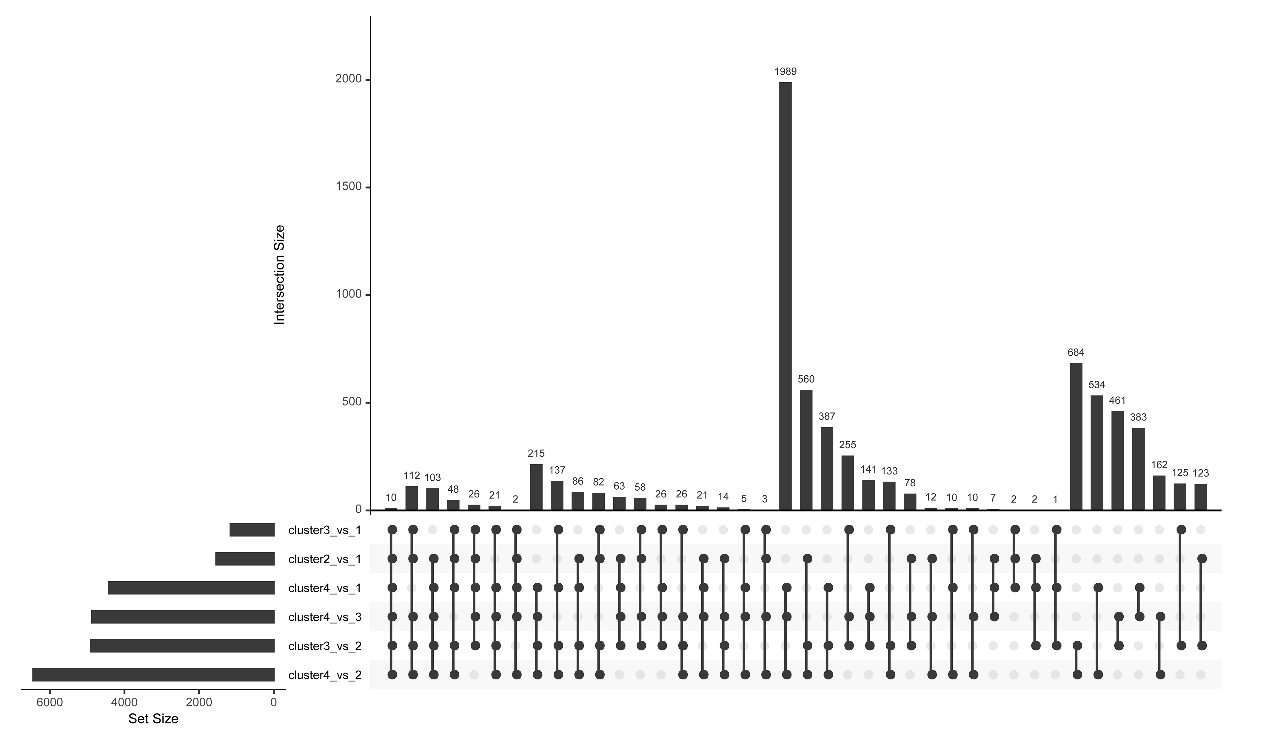


**Fig. S6.** The upset plot showing the number of differently expressed genes (DEGs) of each pairwise groups and the number of intersections of different combinations.


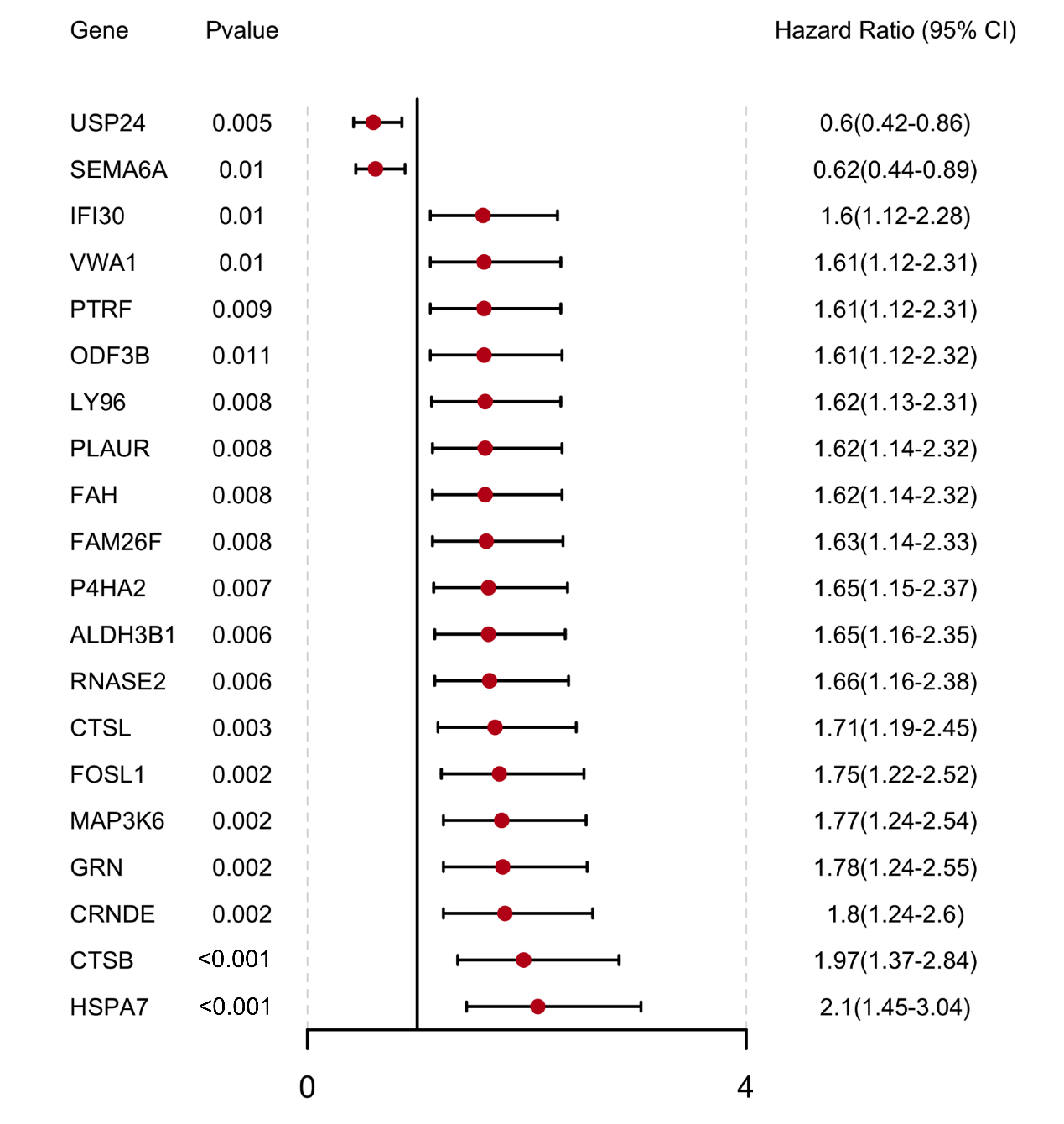


**Fig. S7.** The forest plot presenting the top 20 prognostic differently expressed genes (DEGs) according to the *P* value from small to large.


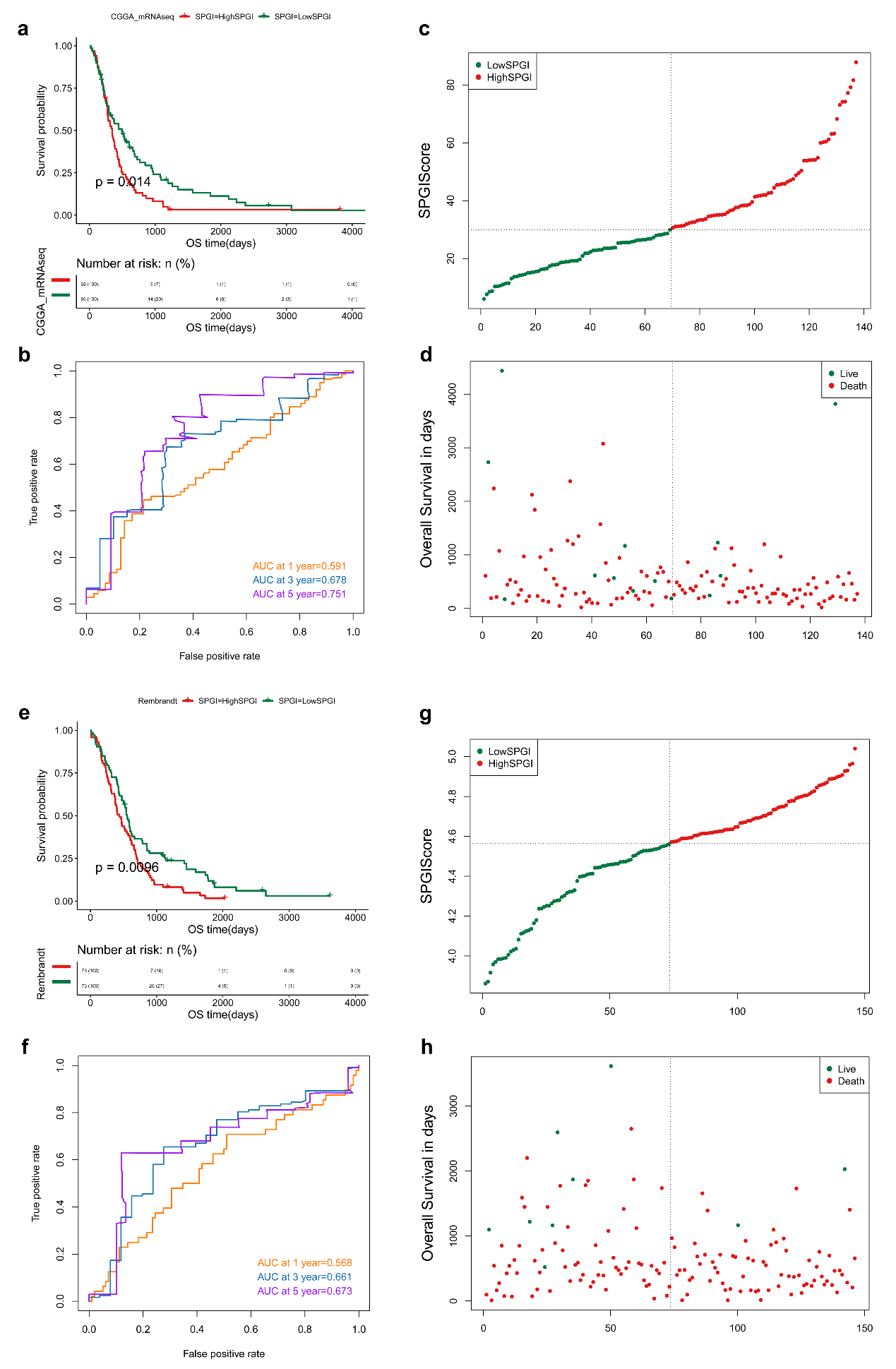


**Fig. S8.** Validation of the sample-specific perturbation of gene interaction score (SPGIScore) in CGGA-mRNAseq_325 cohort and Rembrandt cohort. (a) Kaplan-Meier curve of high- and low-SPGIScore subgroups in CGGA-mRNAseq_325 cohort. (b) ROC curve analysis of SPGIScore in predicting 1-, 3- and 5-year OS in CGGA-mRNAseq_325 cohort. (c-d) The distribution plot of SPGIScore and survival status in CGGA-mRNAseq_325 cohort. (e) Kaplan-Meier curve of high- and low-SPGIScore subgroups in Rembrandt cohort. (f) ROC curve analysis of SPGIScore in predicting 1-, 3- and 5-year OS in Rembrandt cohort. (g-h) The distribution plot of SPGIScore and survival status in Rembrandt cohort.
