# Supplementary material for: Exploring the multidimensional heterogeneities of glioblastoma multiforme based on sample-specific edge perturbation in gene interaction network
Source: Front Immunol. 2022 Aug 29;13:944030. doi: 10.3389/fimmu.2022.944030 (PMC9464945; doi:10.3389/fimmu.2022.944030)
Supplement: Supplementary file 2 [file Table_1.docx]

Supplementary Table S1. The sample number of all cohorts in this study.

| Cohorts | Number of GBM samples | Number of normal brain samples |
| --- | --- | --- |
| TCGA | 155 | - |
| GTEx | - | 1152 |
| CGGA-mRNA-array_301 | 123 | - |
| CGGA-mRNAseq_325 | 137 | - |
| Rembrandt microarray | 146 | - |
