# Supplementary material for: Exploring the multidimensional heterogeneities of glioblastoma multiforme based on sample-specific edge perturbation in gene interaction network
Source: Front Immunol. 2022 Aug 29;13:944030. doi: 10.3389/fimmu.2022.944030 (PMC9464945; doi:10.3389/fimmu.2022.944030)
Supplement: Supplementary file 3 [file Table_2.docx]

**Supplementary Table S2. The primers sequence used in this study.**

| **Name** | **Forward-primer** | **Reverse-primer** |
| --- | --- | --- |
| CRNDE | 5’- TCTGAACTAAGGGGTTCCTCC-3’ | 5’- CCTCCTTCCAATAGCCAGTAC -3’ |
| ANK1 | 5’- CGGCTCGATTGTCTCATACC -3’ | 5’-ACACCAGGACCTTCTCGTACTC-3’ |
| GRN | 5’- CAGTGGGAAGTATGGCTGCT -3’ | 5’- TTAGTGAGGAGGTCCGTGGT -3’ |
| SEMA6A | 5’- GAACTTCATCAAGACGCACCC -3’ | 5’- GGCGGTATCTGACCATTGTTC -3’ |
| β-actin | 5’-TGACGTGGACATCCGCAAAG-3’ | 5’- CTGGAAGGTGGACAGCGAGG-3’ |
