# Supplementary material for: Exploring the multidimensional heterogeneities of glioblastoma multiforme based on sample-specific edge perturbation in gene interaction network
Source: Front Immunol. 2022 Aug 29;13:944030. doi: 10.3389/fimmu.2022.944030 (PMC9464945; doi:10.3389/fimmu.2022.944030)
Supplement: Supplementary file 4 [file Table_3.docx]

**Supplementary Table S3. The primary antibodies used in immunohistochemistry (IHC).**

| **Protein** | **Antibody Name** | **Antibody Number** | **Company** | **Antibody Concentration** |
| --- | --- | --- | --- | --- |
| ANK1 | ANK1 Rabbit pAb | PAB633HU01 | Cloud-Clone Corp | 1:100 |
| GRN | Granulin (GRN) Rabbit pAb | A12440 | ABclonal | 1:200 |
| SEMA6A | SEMA6A Rabbit pAb | CSB-PA875655YA01HU | CUSABIO | 1:100 |
