# Supplementary material for: Exploring the multidimensional heterogeneities of glioblastoma multiforme based on sample-specific edge perturbation in gene interaction network
Source: Front Immunol. 2022 Aug 29;13:944030. doi: 10.3389/fimmu.2022.944030 (PMC9464945; doi:10.3389/fimmu.2022.944030)
Supplement: Supplementary file 5 [file Table_4.docx]

**Supplementary Table S4. Sequences of CRNDE siRNA and GRN siRNA.**

| **Gene** | **Strand** | **Sequence** |
| --- | --- | --- |
| CRNDE | Sense | CCGUUGGUCUUUGAAAUUUTT |
|  | Antisense | AAAUUUCAAAGACCAACGGTT |
| GRN | Sense | GUGUGACCUGAUCCAGAGUAATT |
|  | Antisense | UUACUCUGGAUCAGGUCACACTT |
